# Pneumoperitoneum without significant bowel perforation in patients with blunt trauma: a systematic review and meta-analysis

**DOI:** 10.1186/s13017-026-00673-3

**Published:** 2026-02-01

**Authors:** Emad Masuadi, Yasir Ahmed Mohammed Elhadi, Osman S. Abdelhamed, Zainab M. Alkharas, Linda Östlundh, Gamila Ahmed, Ashraf F. Hefny

**Affiliations:** 1 Epidemiology and Biostatistical Consulting Unit, Institute of Public Health, College of Medicine and Health Sciences, United Arab Emirates University, P.O. Box 15551, Al-Ain, United Arab Emirates; 2 Colorectal Surgery Department, St Vincent’s University Hospital, Dublin, D04 T6F4 Ireland; 3 Family Medicine Department, Dubai Health , Dubai, United Arab Emirates; 4 Örebro University, Örebro, Sweden; 5 Public Services & Outreach Unit, UAEU Library, United Arab Emirates University, P.O. Box 15551, Al-Ain, United Arab Emirates; 6 Department of Surgery, College of Medicine and Health Sciences, United Arab Emirates University, P.O. Box 15551, Al-Ain, United Arab Emirates

**Keywords:** Blunt abdominal trauma, Free intraperitoneal air, Pneumoperitoneum, Hollow viscus injury, Computed tomography, Nontherapeutic laparotomy, Selective nonoperative management, Systematic review and meta-analysis

## Abstract

**Background:**

Free intraperitoneal air (FIA) after blunt trauma is traditionally considered a radiological marker of hollow viscus perforation requiring urgent laparotomy. However, emerging reports have described pneumoperitoneum without surgically meaningful bowel injury, raising concerns about unnecessary operations. This systematic review and meta-analysis aimed to quantify the proportion of patients with blunt trauma with computed tomography (CT)-detected FIA who had no significant bowel perforation, defined as either (1) non-therapeutic laparotomy with no identified macroscopic perforation or (2) successful nonoperative management without subsequent clinical deterioration.

**Methods:**

This review followed the 2020 Preferred Reporting Items for Systematic Reviews and Meta-Analyses guidelines and was prospectively registered in the International Prospective Register of Systematic Reviews (CRD42020202174). PubMed, Embase, Scopus, and Web of Science were searched through November 13, 2025, for observational studies reporting the outcomes of patients with blunt trauma with CT-detected FIA. Two reviewers independently performed study selection, data extraction, and quality assessment using the Newcastle–Ottawa Scale. A random-effects meta-analysis was performed to estimate the pooled proportion of FIA cases without significant perforation. Heterogeneity was assessed using I-squared statistic (measure of heterogeneity) and τ^2^; small-study effects were examined using contour-enhanced funnel plots and Egger’s regression. Case reports meeting eligibility criteria were narratively summarized.

**Results:**

Fourteen studies comprising 8,972 patients with blunt trauma were included. Among them, 239 (2.7%) had CT-detected FIA. In the FIA subgroup, 117 patients (49.0%) had surgically confirmed bowel perforation, whereas 122 (51.0%) had no significant perforation, defined as a non-therapeutic laparotomy or a stable nonoperative clinical course. Among patients without FIA on CT, 56 of 8,733 (0.6%) had bowel perforation identified during surgery. The pooled analysis showed that 34% (95% CI 14–63%) of patients with FIA had no significant perforation. Substantial heterogeneity was observed (I-squared statistic = 80.3%, τ^2^ = 3.26, *p* < 0.001), reflecting variations in CT acquisition, diagnostic criteria, and operative thresholds. Funnel plot asymmetry suggested potential small-study effects. Additionally, 19 case reports (20 patients) published between 1999 and 2025 illustrated that benign pneumoperitoneum most often occurred in young men following high-energy trauma, commonly associated with pneumothorax or pneumomediastinum; most underwent nontherapeutic laparotomy, whereas several were successfully managed nonoperatively.

**Conclusion:**

A noteworthy subgroup of patients with blunt trauma with CT-detected FIA did not exhibit clinically significant bowel perforations. Although FIA remains an important radiologic warning sign, it is not an independent diagnostic indicator of significant hollow viscus injury. Clinical decision-making should integrate clinical assessment with adjunctive CT findings rather than rely on FIA alone. Owing to the rarity of FIA and limited sample sizes, larger prospective studies are required to refine the diagnostic performance of FIA and optimize selective nonoperative management strategies.

**Supplementary Information:**

The online version contains supplementary material available at 10.1186/s13017-026-00673-3.

## Background

Free intraperitoneal air (FIA) following blunt trauma is traditionally regarded as a critical radiological finding that raises immediate concerns regarding hollow viscus perforation and often prompts urgent laparotomy. Although bowel gas is normally confined to the gastrointestinal lumen, traumatic forces may allow air to escape into the peritoneal cavity. Prompt recognition of clinically significant bowel injuries is essential because delayed intervention is associated with increased morbidity and mortality [1]. However, FIA is not uniformly synonymous with surgically relevant perforations. Air may reach the peritoneal cavity through nonenteric pathways—including dissection of thoracic air, barotrauma related to mechanical ventilation, or gynecologic sources—or may arise from minor, self-limited bowel wall disruptions that do not require operative intervention [2, 3].

Computed tomography (CT) is the diagnostic modality of choice for evaluating blunt abdominal trauma. Several CT features, such as bowel wall thickening, mesenteric hematoma, and unexplained free fluid, are well-established predictors of bowel and mesenteric injuries [4, 5]. FIA, while an important warning sign, has demonstrated limited sensitivity and positive predictive value for diagnosing clinically significant perforation, with isolated pneumoperitoneum sometimes occurring in the absence of bowel injury [6, 7]. Small volumes of air, particularly when unaccompanied by other CT abnormalities, may therefore represent a benign entity rather than a marker of full-thickness perforation. Importantly, evidence from surgical series and case reports shows that some patients with blunt trauma with FIA do not have surgically demonstrable bowel perforation during laparotomy, whereas others remain clinically stable under nonoperative management [8]. While FIA warrants vigilance, treating all cases as perforations may expose patients to nontherapeutic laparotomy, which carries substantial morbidity, increased length of stay, and higher healthcare costs without improving survival [9].

Despite the growing recognition of benign traumatic pneumoperitoneum, the actual proportion of patients with blunt trauma with CT-detected FIA who do not have clinically significant bowel perforations remains unclear. Existing evidence is fragmented across heterogeneous observational studies and isolated case reports, and terminology varies widely, leading to inconsistencies in reporting and interpretation. To our knowledge, no previous systematic review has synthesized this evidence, quantified the pooled proportion of FIA cases without significant perforation, or summarized the potential benign mechanisms underlying FIA in blunt trauma. Therefore, this study aimed to systematically identify and synthesize all available data to (1) estimate the pooled proportion of patients with blunt trauma with CT-confirmed FIA who had no significant bowel perforation, defined as either negative laparotomy findings or successful nonoperative management without clinical deterioration, and (2) narratively summarize the reported benign mechanisms of traumatic pneumoperitoneum.

## Materials and methods

### Study design and literature search

This systematic review and meta-analysis was conducted in accordance with the Preferred Reporting Items for Systematic Reviews and Meta-Analyses 2020 guidelines [10] and prospectively registered in International Prospective Register of Systematic Reviews (CRD42020202174) [11]. A comprehensive search of PubMed, Embase, Scopus, and Web of Science was first performed from database inception to November 13, 2024, and subsequently updated during manuscript revision to include all studies published through November 2025, owing to the rarity of traumatic FIA. Search strategies combined Medical Subject Headings and keywords related to blunt trauma, bowel perforation, and FIA, with English-language restrictions applied. The full search strategies are provided in the Supplementary Material. The reference lists of included articles were screened to identify additional eligible studies.

### Eligibility criteria

We included peer-reviewed observational studies (prospective or retrospective) reporting the outcomes of adult patients with blunt trauma with CT-detected FIA, in which patients were managed operatively or nonoperatively and the final bowel injury status could be determined. Studies were excluded if they met any of the following criteria:

1. Included penetrating trauma, pediatric populations, or nontraumatic pneumoperitoneum.
2. Lacked CT confirmation of FIA.
3. Did not report whether bowel perforation was confirmed or excluded.
4. Were review articles, commentaries, or conference abstracts.

Case reports and small case series were excluded from the meta-analysis but were synthesized narratively because of their relevance in describing benign traumatic pneumoperitoneum.

### Screening and study selection

The search results were imported into Covidence (Veritas Health Innovation, Melbourne, Australia) for automated and manual duplicate removal. Two reviewers (YAME and OS), who were blinded to each other’s decisions, screened titles and abstracts according to the predefined eligibility criteria. The full texts of potentially eligible studies were independently assessed. Discrepancies were resolved by discussion or consultation with a third reviewer (AFH).

### Data extraction and risk of bias assessment

Two reviewers (YAME and OS) independently extracted data using a standardized extraction sheet. Extracted variables included study design, sample size, mechanisms of injury, number of FIA cases, operative findings, nonoperative outcomes, and presence of bowel perforation. Any disagreements were resolved by consensus.

The risk of bias for observational studies was assessed independently by two reviewers using the Newcastle–Ottawa Scale [12]. Total scores ranged from five to seven of nine, indicating moderate methodological quality. Most studies demonstrated adequate case ascertainment and outcome verification, although comparability scores were consistently low because of limited adjustment for confounders. Full quality assessment results are provided in the Supplementary Material.

### Operational definition

Given the variable terminology across studies, we adopted a standardized definition of no significant bowel perforation to ensure consistency. This category included:

1. Nontherapeutic laparotomy, defined as the absence of a macroscopic full-thickness perforation despite CT-detected FIA. This definition acknowledges that minor or microscopic injuries may not be visible but are not surgically consequential.
2. Successful nonoperative management, defined as hemodynamic and clinical stability during observation, with follow-up clinical assessment and/or imaging showing no deterioration, no delayed perforation, and no need for operative intervention.

This definition aligns with prior trauma literature. Hefny et al. [4] classified conservatively managed FIA as false-positive for perforation, whereas Marek et al. [5] categorized both negative laparotomy and stable nonoperative management outcomes as “benign free air.”

### Synthesis and analysis

The primary outcome was the pooled proportion of patients with blunt trauma with CT-detected FIA who had no significant bowel perforation using the operational definition above. A meta-analysis of proportions was performed using a random-effects generalized linear mixed model with a logit link. This approach provides robust estimation of sparse and heterogeneous data and avoids biases associated with traditional transformation methods. Studies reporting zero events were retained using a minimal continuity correction (0.5 added to zero-event studies), consistent with Cochrane guidelines for rare-event meta-analyses. Heterogeneity was quantified using τ^2^ and I-squared statistic (measure of heterogeneity) (I^2^). Potential small-study effects were explored using contour-enhanced funnel plots and Egger regression tests. Case reports were not included in the meta-analysis but were summarized narratively to provide qualitative insight into benign mechanisms of pneumoperitoneum and patterns of conservative versus operative management.

## Results

**Fig. 1:**
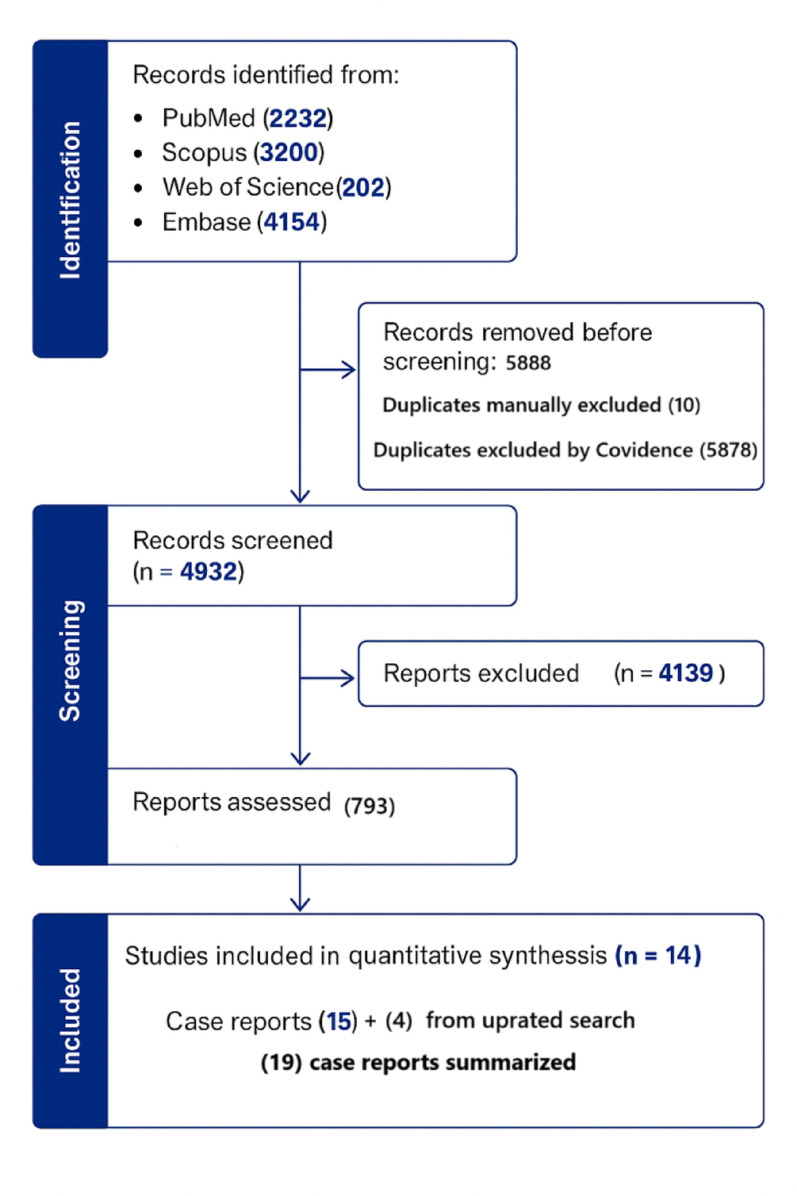
Preferred reporting items for systematic reviews and meta-analyses flow diagram of included studies

The initial search identified 10,820 studies across the four databases. After removing 5,888 duplicates (5,878 via Covidence and 10 manually), 4,932 studies remained. Of these, 4,139 were excluded based on titles and abstracts, leaving 793 studies for full-text review. Further assessment excluded 779 studies, comprising 15 case reports and 764 irrelevant studies. The updated search yielded no additional original studies; however, four newly published case reports meeting the inclusion criteria were identified. Finally, 14 studies met the inclusion criteria and were included in the quantitative synthesis (Fig. 1).

### Summary of included studies

Table 1 presents the characteristics of the 14 studies included in the meta-analysis. Most studies were retrospective in design, with only two studies (Ku et al. [13] and Surmalbhai et al. [14]) conducted prospectively. Most cases of blunt trauma resulted from road traffic collisions or falls from heights. The number of patients with CT-confirmed FIA varied widely across studies, ranging from as few as four (Breen et al. [7]) to as many as 76 (Bhagvan et al. [15]). Several studies reported that a high proportion of patients underwent laparotomy; however, the proportion of patients with FIA who had no significant bowel perforation varied considerably. Studies, such as Breen et al. [7], Rizzo et al. [16], Tan et al. [17], and Hagiwara et al. [18], did not report any cases of FIA without significant perforation during laparotomy. Conversely, Hamilton et al. [19], Marek et al. [5], and Hefny et al. [4] documented a substantial proportion of patients with FIA without significant bowel perforation.

**Table 1:** Summary of included studies and patients’ findings (N = 14 studies, 8,972 patients)

Study	Study design	N trauma patients with CT scan	N CT scan confirmed FIA	N FIA and perforation at laparotomy	N FIA without detectable perforation at laparotomy	Successful nonoperative management	N FIA and no perforation at laparotomy or successful nonoperative management	N without FIA on CT scan and perforation at laparotomy
Bohmer and Cowan [20]	Retrospective	116	11	7	1	3	4	2
Breen et al. [7]	Retrospective	31	4	4	0	0	0	5
Firetto et al. [21]	Retrospective	831	12	10	0	2	2	
Rizzo et al. [16]	Retrospective	51	9	9	0	0	0	1
Tan et al. [17]	Retrospective	41	11	11	0	0	0	5
Marek et al. [5]	Retrospective	5877	74	16	24	34	58	
Hagiwara et al. [18]	Retrospective	430	6	6	0	0	0	7
Ku et al. [13]	Prospective	394	22	15	7	0	7	9
Bhagvan et al. [15]	Retrospective	78	9	5	4	0	4	8
Hamilton et al. [19]	Retrospective	118	7	0	2	5	7	0
Kane et al. [22]	Retrospective		18	4	2	12	14	
Hefny et al. [4]	Retrospective	419	21	2	0	19	19	2
Faget et al. [23]	Retrospective	556	21	15	6	0	6	12
Surmalbhai et al. [14]	Prospective	30	14	13	1	0	1	5
Total	8972	239	117	47	75	122	56	

CT, computed tomography; FIA, free intraperitoneal air

**Fig. 2:**
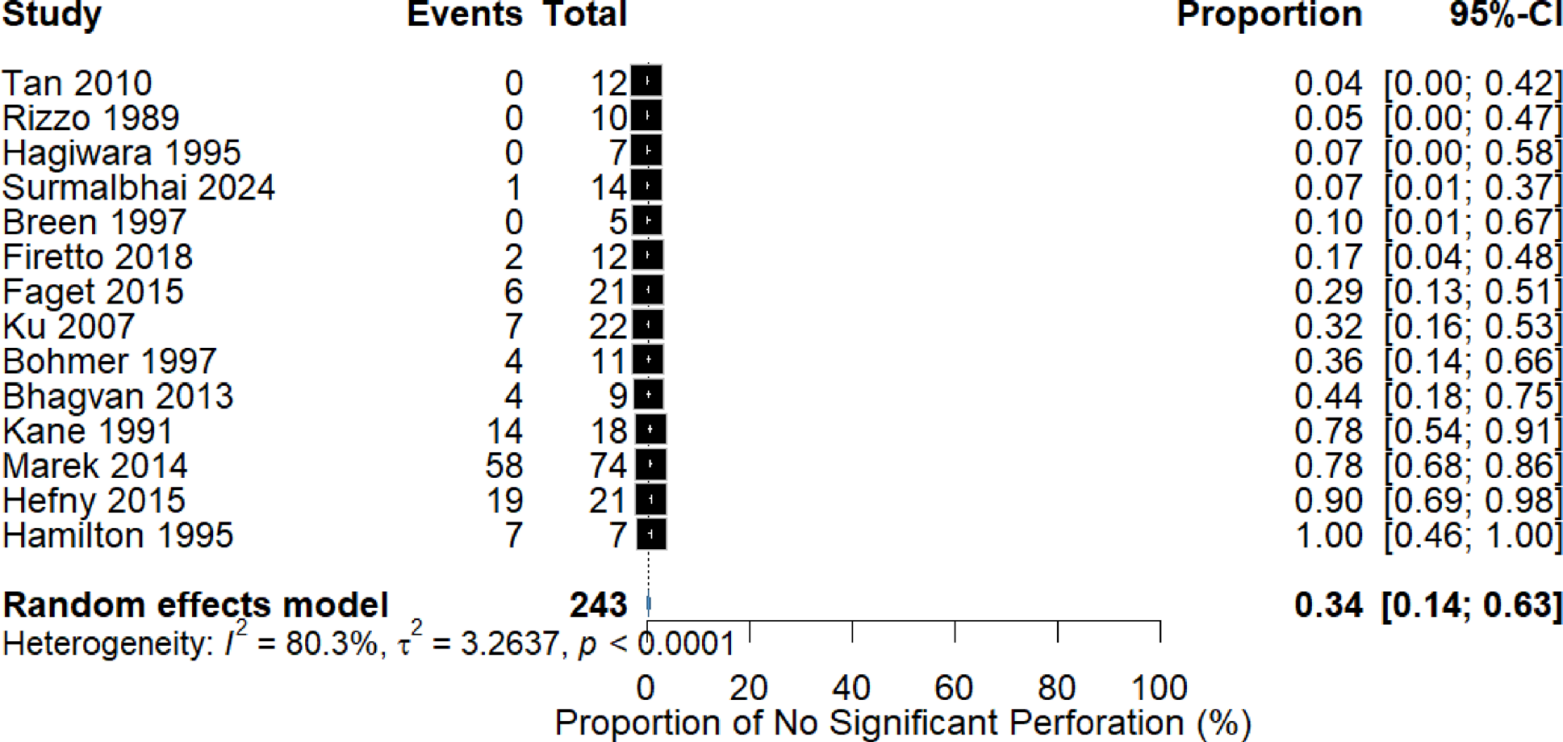
Forest plot showing the pooled proportion of patients with blunt trauma with computed tomography-detected free intraperitoneal air who had no significant bowel perforation, defined as either negative laparotomy or successful nonoperative management

Across all studies, 239 patients (2.7%) had CT-confirmed FIA. Among these patients, 117 (49.0%) had confirmed bowel perforation during laparotomy, whereas 122 (51.0%) had no significant perforation, defined as negative laparotomy (n = 47) or successful nonoperative management without subsequent deterioration (n = 75). By comparison, among patients without FIA on CT, 56 of 8,733 (0.6%) were found to have bowel perforation at surgery. Using a random-effects meta-analysis with maximum likelihood estimation and Hartung–Knapp adjustment, the pooled proportion of patients with FIA who had no significant perforation was 34% (95% CI: 14%–63%) (Fig. 2). Substantial between-study heterogeneity was observed (I^2^ = 80.3%, τ^2^ = 3.2637, *p* < 0.0001), reflecting methodological and clinical variation across cohorts. Funnel plot inspection revealed an asymmetric distribution, suggesting potential small-study effects (Supplementary Material).

### Narrative synthesis of case reports

In addition to the quantitative synthesis, we identified 19 published case reports describing 20 patients with FIA following blunt trauma who had no significant bowel perforation according to our operational definition. These cases provide additional clinical context regarding the spectrum of benign pneumoperitoneum in trauma and highlight important variations in diagnostic pathways and management decisions. Most patients were men (84%) and young to middle-aged, with mechanisms of injury dominated by road traffic collisions, although several cases resulted from falls from heights or assault. Associated thoracic findings, such as pneumothorax or pneumomediastinum, were reported in multiple cases, supporting the hypothesis that extra-abdominal air can dissect into the peritoneal cavity without bowel disruption.

Laparotomy was performed in 15 of 20 patients, most of whom demonstrated no intraoperative evidence of bowel perforation, whereas five patients were successfully managed nonoperatively with clinical observation and, when reported, follow-up imaging demonstrating stability. A detailed summary of individual case characteristics, including patient demographics, mechanism of injury, associated thoracic air, diagnostic pathways, operative versus nonoperative management, and final outcomes, is presented in Table 2.

**Table 2:** Summary of case reports of benign free air in patients with blunt trauma

Study	Age/Sex	Blunt trauma mechanism	Cases	Study type	Associated Px or Pm	Laparotomy	Nonoperative management
Gardner-T and Maddox [24]	34/M	RTC	1	Case report	1	1	0
Nishina et al. [25]	60/M	Fall from height	1	Case report	1	0	1
Mussack et al. [26]	47/M	Fall from height	1	Letter to editor	1	1	0
Assenza et al. [27]	21/M	RTC	1	Case report	1	1	0
Di Saverio et al. [28]	21/M & 20/M	Assault and TRC	2	Case report	2	1	1
Hakim et al. [29]	45/M	RTC	1	Case report	1	1	0
Webman et al. [30]	56/M	RTC	1	Case report	1	1	0
Curfman et al. [31]	82/M	Fall of stairs	1	Case report	1	1	0
Carzolio et al. [32]	21/M	RTC	1	Case report	1	0	1
Lebby et al. [33]	37/M	RTC	1	Case report	0	1	0
Castro et al. [34]	78/M	RTC	1	Case report	1	1	0
Ubukata et al. [35]	95/M	RTC	1	Case report	0	1	0
Parvez et al. [36]	27/M	RTC	1	Case report	0	1	0
Kang and Choi [37]	45/F	RTC	1	Case report	0	1	0
Goyal et al. [38]	22/M	RTC	1	Case report	1	1	0
Lu et al. [39]	16/F	Fall from height	1	Case report	0	1	0
Haddar et al. [40]	46/M	Fall from height	1	Case report	0	0	1
Karoui et al. [41]	60/M	RTC	1	Case report	0	1	0
Rai et al. [42]	56/M	RTC	1	Case report	0	1	0

Associated Px or Pm**:** associated pneumothorax or pneumomediastinum; RTC: road traffic collision

## Discussion

The findings of this systematic review and meta-analysis indicate that an identifiable subgroup of patients with blunt trauma with CT-detected FIA does not have clinically significant bowel perforation. This challenges the long-standing assumption that intraperitoneal air invariably requires surgical exploration. The pooled estimate of 34% (95% CI: 14%–63%) demonstrates considerable uncertainty but confirms that isolated FIA is not uniformly indicative of a hollow viscus injury requiring surgery. The wide confidence intervals and high heterogeneity underscore that these results must be interpreted with caution.

The substantial heterogeneity observed (I^2^ = 80.3%, τ^2^ = 3.26) likely reflects genuine clinical variation across studies, including differences in CT protocols, injury mechanisms, criteria for selecting patients for laparotomy, and definitions of clinically significant perforation. Importantly, modern multidetector CT scanners are highly sensitive for detecting even very small volumes of intraperitoneal air, including air of nonenteric origin. This increased sensitivity, while valuable for injury detection, may also lead to false-positive interpretations for bowel injury, thereby increasing the risk of nontherapeutic laparotomy.

Earlier studies, particularly those conducted before modern CT technology became widespread, frequently regarded FIA as pathognomonic for bowel perforation and therefore recommended mandatory exploratory laparotomy. Marek et al. identified a relatively high proportion of benign or clinically insignificant free air and advocated selective operative decision-making [5]. These more contemporary findings align with our pooled estimate and support selective nonoperative approaches in carefully evaluated patients.

Several nonenteric mechanisms may explain benign FIA in patients with blunt trauma. These include air tracking from pneumothorax or pneumomediastinum via the Macklin effect [43], barotrauma from positive-pressure ventilation, transiently sealed microscopic perforations, pseudopneumoperitoneum, and, more rarely, gynecologic sources. Therefore, FIA alone on CT may be an unreliable indicator of clinically significant bowel injury. However, adjunctive CT findings, such as unexplained free fluid, bowel wall discontinuity, mesenteric hematoma or stranding, or extraluminal contrast, substantially increase diagnostic confidence for clinically significant perforation [44]. Additionally, the number, size, and distribution of air pockets may help clinically differentiate pathologic perforation-related air from benign air patterns [4].

A narrative synthesis of 19 published case reports (20 patients) further reinforces these findings. Most patients were young men who sustained high-energy blunt trauma commonly associated with thoracic injuries, and none had a surgically meaningful bowel perforation. Among these patients, 15 underwent laparotomy with negative findings, whereas five were successfully managed nonoperatively. These reports highlight the risk of unnecessary surgery when FIA is considered in isolation and support a more selective, clinically integrated management strategy. Our analysis also showed that 0.6% of patients had bowel perforation despite no FIA on CT, consistent with the findings of prior literature demonstrating that CT sensitivity, although high, is not absolute. For patients with equivocal or nonspecific CT findings but high clinical suspicion, selective nonoperative management involving close observation, serial abdominal examinations, and, when indicated, repeat imaging remains a safe and evidence-based approach [8, 45]. Large trauma registry studies have further shown that nontherapeutic laparotomies are associated with increased morbidity and postoperative complications [8], reinforcing the need for careful patient selection.

Overall, the present findings support a structured, selective diagnostic algorithm that integrates clinical assessment with radiologic features rather than relying solely on the presence of FIA [5]. Surgical intervention should be reserved for patients with corroborative CT findings, clinical signs of peritonitis, or hemodynamic instability. Further research is needed to develop standardized CT-based scoring systems, define optimal monitoring protocols, and quantify the cost–benefit impact of reducing nontherapeutic laparotomies.

### Limitations

This study had several limitations. First, verification bias is inherent, as not all patients with FIA underwent laparotomy. In some cases, “no significant perforation” was inferred from stable nonoperative management, which may underestimate the presence of macroscopic bowel perforation. Definitions of FIA, perforation, and required duration of observation varied across studies, introducing heterogeneity despite the use of a standardized operational definition. The predominance of retrospective, single-center studies further limits control over confounding factors and reduces the comparability of management thresholds. Additionally, FIA is a rare finding, accounting for 2.7% of CT-imaged trauma patients, resulting in small event counts and wide confidence intervals. Assessment of publication bias is also constrained, as funnel plots and related tests are not well calibrated for rare-event proportion meta-analyses. In addition, although the narrative synthesis of case reports provides practical clinical context, such reports are inherently subject to substantial publication bias, including selective reporting of atypical or benign presentations. This limitation may inadvertently over-weight anecdotal benign cases and restrict the generalizability of findings derived from case-level evidence.

## Conclusions

A noteworthy subgroup of patients with blunt trauma with CT-detected FIA did not show clinically significant bowel perforation. Although FIA remains an important radiologic warning sign, it is not an independent diagnostic indicator of significant hollow viscus injury. Decision-making should integrate clinical assessment with adjunctive CT findings rather than rely on FIA alone. Owing to the rarity of FIA and limited sample sizes, larger prospective studies are required to refine the diagnostic performance of FIA and optimize selective nonoperative management strategies.

## Supplementary Information

Below is the link to the electronic supplementary material.

Supplementary Material 1

## Data Availability

All data supporting the findings of this study are available from the corresponding author upon request.

## Abbreviations

FIA: Free intraperitoneal air
CT: Computed tomography
I^2^: I-squared statistic (measure of heterogeneity)
