# Supplementary material for: Pneumoperitoneum without significant bowel perforation in patients with blunt trauma: a systematic review and meta-analysis
Source: World J Emerg Surg. 2026 Feb 1;21:11. doi: 10.1186/s13017-026-00673-3 (PMC12952182; doi:10.1186/s13017-026-00673-3)
Supplement: Supplementary file 1 — Supplementary Material 1 [file 13017_2026_673_MOESM1_ESM.docx]

**Table S1 Literature search strategy**

| **Source** | **Search string** | **Results** | **Notes** |
| --- | --- | --- | --- |
| **PubMed**  **Coverage:**  From database inception to search date  **Search date:**  2024-11-13 | ("blunt trauma*"[Title/Abstract] OR "trauma, blunt"[Title/Abstract] OR "nonpenetrating wound*"[Title/Abstract] OR "wound, nonpenetrating"[Title/Abstract] OR "injuries, nonpenetrating"[Title/Abstract] OR "nonpenetrating injur*"[Title/Abstract] OR "injury, nonpenetrating"[Title/Abstract] OR "injuries, blunt"[Title/Abstract] OR "blunt injur*"[Title/Abstract] OR "injury, blunt"[Title/Abstract] OR "blunt abdominal trauma"[Title/Abstract] OR "blunt abdominal injur*"[Title/Abstract] OR "blunt chest trauma"[Title/Abstract] OR "blunt chest injur*"[Title/Abstract] OR "blunt pelvic trauma"[Title/Abstract] OR "blunt pelvic injur*"[Title/Abstract] OR "blunt bowel trauma"[Title/Abstract] OR "blunt bowel injur*"[Title/Abstract] OR "blunt mesentery trauma"[Title/Abstract] OR "blunt mesentery injur*"[Title/Abstract] OR "blunt intestines trauma"[Title/Abstract] OR "blunt intestines injur*"[Title/Abstract] OR "blunt gastrointestinal trauma"[Title/Abstract] OR "blunt gastrointestinal injur*"[Title/Abstract] OR "blunt thoracic trauma"[Title/Abstract] OR "blunt thoracic injur*"[Title/Abstract] OR "blunt cervical trauma"[Title/Abstract] OR "blunt cervical injur*"[Title/Abstract] OR "blunt forced trauma"[Title/Abstract] OR "blunt forced injur*"[Title/Abstract] OR "bowel perforation*"[Title/Abstract] OR "intestinal perforation*"[Title/Abstract] OR "enteric perforation*"[Title/Abstract] OR "gastrointestinal perforation*"[Title/Abstract] OR "Wounds, Nonpenetrating"[Mesh:NoExp] OR "Intestinal Perforation"[Mesh]) AND ("pneumoperitoneum"[Title/Abstract] OR air[Title/Abstract] OR "Pneumoperitoneum"[Mesh]) | [2,232](https://pubmed.ncbi.nlm.nih.gov/?term=longquerye3dc456df80e41e12610&sort=) | All terms are searched in the search fields “title” and “abstract” (here marked with TI/AB) and in MeSH (when available).  A filter for English language is applied. |
| **Scopus**  **Coverage:**  From database inception to search date  **Search date:**  2024-11-13 | ( TITLE-ABS-KEY ( "blunt trauma*" ) OR TITLE-ABS-KEY ( "trauma, blunt" ) OR TITLE-ABS-KEY ( "nonpenetrating wound*" ) OR TITLE-ABS-KEY ( "wound, nonpenetrating" ) OR TITLE-ABS-KEY ( "injuries, nonpenetrating" ) OR TITLE-ABS-KEY ( "nonpenetrating injur*" ) OR TITLE-ABS-KEY ( "injury, nonpenetrating" ) OR TITLE-ABS-KEY ( "injuries, blunt" ) OR TITLE-ABS-KEY ( "blunt injur*" ) OR TITLE-ABS-KEY ( "injury, blunt" ) OR TITLE-ABS-KEY ( "blunt abdominal trauma" ) OR TITLE-ABS-KEY ( "blunt abdominal injur*" ) OR TITLE-ABS-KEY ( "blunt chest trauma" ) OR TITLE-ABS-KEY ( "blunt chest injur*" ) OR TITLE-ABS-KEY ( "blunt pelvic trauma" ) OR TITLE-ABS-KEY ( "blunt pelvic injur*" ) OR TITLE-ABS-KEY ( "blunt bowel trauma" ) OR TITLE-ABS-KEY ( "blunt bowel injur*" ) OR TITLE-ABS-KEY ( "blunt mesentery trauma" ) OR TITLE-ABS-KEY ( "blunt mesentery injur*" ) OR TITLE-ABS-KEY ( "blunt intestines trauma" ) OR TITLE-ABS-KEY ( "blunt intestines injur*" ) OR TITLE-ABS-KEY ( "blunt gastrointestinal trauma" ) OR TITLE-ABS-KEY ( "blunt gastrointestinal injur*" ) OR TITLE-ABS-KEY ( "blunt thoracic trauma" ) OR TITLE-ABS-KEY ( "blunt thoracic injur*" ) OR TITLE-ABS-KEY ( "blunt cervical trauma" ) OR TITLE-ABS-KEY ( "blunt cervical injur*" ) OR TITLE-ABS-KEY ( "blunt forced trauma" ) OR TITLE-ABS-KEY ( "blunt forced injur*" ) OR TITLE-ABS-KEY ( "bowel perforation*" ) OR TITLE-ABS-KEY ( "intestinal perforation*" ) OR TITLE-ABS-KEY ( "enteric perforation*" ) OR TITLE-ABS-KEY ( "gastrointestinal perforation*" ) ) AND ( TITLE-ABS-KEY ( "pneumoperitoneum" ) OR TITLE-ABS-KEY ( "air" ) ) | [3,200](https://www-scopus-com.uaeu.idm.oclc.org/search/history/results.uri?origin=searchhistory&shid=2) | All terms are searched in the search fields “title”, “abstract” and “keywords”.  No thesaurus available.  A filter for English language is applied. |
| **Web of Science**  (Core Collection)  **Coverage:** From database inception to search date  **Search date:**  2024-11-13 | **#1**  (((((((((((((((((((((((((((((((((TS=("blunt trauma*” )) OR TS=("trauma, blunt" )) OR TS=("nonpenetrating wound*" )) OR TS=("wound, nonpenetrating” )) OR TS=("injuries, nonpenetrating" )) OR TS=("nonpenetrating injur*")) OR TS=("injury, nonpenetrating" )) OR TS=("injuries, blunt" )) OR TS=("blunt injur*" )) OR TS=("injury, blunt" )) OR TS=("blunt abdominal trauma" )) OR TS=("blunt abdominal injur*" )) OR TS=("blunt chest trauma" )) OR TS=("blunt chest injur*" )) OR TS=("blunt pelvic trauma" )) OR TS=("blunt pelvic injur*" )) OR TS=("blunt bowel trauma" )) OR TS=("blunt bowel injur*" )) OR TS=("blunt mesentery trauma" )) OR TS=("blunt mesentery injur*" )) OR TS=("blunt intestines trauma" )) OR TS=("blunt intestines injur*" )) OR TS=("blunt gastrointestinal trauma" )) OR TS=("blunt gastrointestinal injur*" )) OR TS=("blunt thoracic trauma” )) OR TS=("blunt thoracic injur*")) OR TS=("blunt cervical trauma")) OR TS=("blunt cervical injur*" )) OR TS=("blunt forced trauma")) OR TS=("blunt forced injur*" )) OR TS=("bowel perforation*" )) OR TS=("intestinal perforation*" )) OR TS=("enteric perforation*" )) OR TS=("gastrointestinal perforation*")  <https://www.webofscience.com/wos/woscc/summary/faecf217-b4db-4441-af28-53b4432bbe90-011eab5c10/relevance/1>  **#2 (TS=("pneumoperitoneum" )) OR TS=( air)**  <https://www.webofscience.com/wos/woscc/summary/11cfe16f-3728-4a32-af7b-cd91ddc88921-011eab9bf4/relevance/1>  **#1 & #2** <https://www-webofscience-com.uaeu.idm.oclc.org/wos/woscc/summary/f0745ec3-f099-4b3d-9d25-d5d4246f9ae2-012c594cb7/relevance/1> | [1234](https://www-webofscience-com.uaeu.idm.oclc.org/wos/woscc/summary/f0745ec3-f099-4b3d-9d25-d5d4246f9ae2-012c594cb7/relevance/1) | All terms are searched in the field “topic” (including title, abstract and author supplied keywords, here marked with “TOPIC”).  No thesaurus available.  A filter for English language is applied. |
| **Embase**  **Source**: Embase and Medline  **Coverage:**  From database inception to search date  **Search date:**  2024-11-13 | ('blunt trauma':ab,ti OR 'blunt traumas':ab,ti OR 'trauma, blunt':ab,ti OR 'nonpenetrating wound':ab,ti OR 'nonpenetrating wounds':ab,ti OR 'wound, nonpenetrating':ab,ti OR 'injuries, nonpenetrating':ab,ti OR 'nonpenetrating injuries':ab,ti OR 'injury, nonpenetrating':ab,ti OR 'nonpenetrating injury':ab,ti OR 'injuries, blunt':ab,ti OR 'blunt injury':ab,ti OR 'injury, blunt':ab,ti OR 'blunt injuries':ab,ti OR 'blunt abdominal trauma':ab,ti OR 'blunt abdominal injury':ab,ti OR 'blunt abdominal injuries':ab,ti OR 'blunt chest trauma':ab,ti OR 'blunt chest injury':ab,ti OR 'blunt chest injuries':ab,ti OR 'blunt pelvic trauma':ab,ti OR 'blunt pelvic injury':ab,ti OR 'blunt pelvic injuries':ab,ti OR 'blunt bowel trauma':ab,ti OR 'blunt bowel injury':ab,ti OR 'blunt bowel injuries':ab,ti OR 'blunt mesentery trauma':ab,ti OR 'blunt mesentery injury':ab,ti OR 'blunt mesentery injuries':ab,ti OR 'blunt intestines trauma':ab,ti OR 'blunt intestines injury':ab,ti OR 'blunt intestines injuries':ab,ti OR 'blunt gastrointestinal trauma':ab,ti OR 'blunt gastrointestinal injury':ab,ti OR 'blunt gastrointestinal injuries':ab,ti OR 'blunt thoracic trauma':ab,ti OR 'blunt thoracic injury':ab,ti OR 'blunt thoracic injuries':ab,ti OR 'blunt cervical trauma':ab,ti OR 'blunt cervical injury':ab,ti OR 'blunt cervical injuries':ab,ti OR 'blunt forced trauma':ab,ti OR 'blunt forced injury':ab,ti OR 'blunt forced injuries':ab,ti OR 'bowel perforation':ab,ti OR 'bowel perforations':ab,ti OR 'intestinal perforation':ab,ti OR 'intestinal perforations':ab,ti OR 'enteric perforation':ab,ti OR 'enteric perforations':ab,ti OR 'gastrointestinal perforation':ab,ti OR 'gastrointestinal perforations':ab,ti OR 'blunt trauma'/exp OR 'blunt trauma' OR 'intestine perforation'/exp OR 'intestine perforation') AND ('pneumoperitoneum':ab,ti OR air:ab,ti OR 'pneumoperitoneum'/exp OR 'pneumoperitoneum') | 4,154 | All terms are searched in the fields: “title” and “abstract” (here marked with “:ab,ti”) and in the “thesaurus” (here marked with “/de or /exp”) when available.  A filter for English language is applied.  Thesauru (Emtree) variations compared to PubMed’s MeSH is applied as per availability and recommendation Embase. |
| **Total no. of references identified** | | | **10,820** |
| **Total no. of references after de-duplication** | | | **4,932** |

The search has been updated using the same strategy during the manuscript review stage to cover the period up to November 2025 and 4 case reports were added and summarized.

**Table S2.** Quality assessment of retrospective studies using the Newcastle-Ottawa scale

| First author / year | Selection | | | | Comparability | | Outcome | | | Score |
| --- | --- | --- | --- | --- | --- | --- | --- | --- | --- | --- |
|  | Item 1 | Item 2 | Item 3 | Item 4 | Item 5 | Item 6 | Item 7 | Item 8 | Item 9 |  |
| Bohmer / 1997 | * | - | * | * | - | * | * | * | * | 7 |
| Breen / 1997 | * | * | * | - | - | - | * | * | - | 6 |
| Firetto / 2018 | * | - | * | - | - | - | * | * | * | 5 |
| Rizzo /1989 | * | - | * | - | - | - | * | * | * | 5 |
| Tan / 2010 | * | - | * | - | - | - | * | * | * | 5 |
| Marek / 2014 | * | * | * | - | * | - | * | * | * | 7 |
| Hagiwara / 1995 | * | - | * | - | - | - | * | * | * | 5 |
| Ku / 2007 | * | - | * | * | - | - | * | * | * | 6 |
| Bhagvan / 2013 | * | - | * | - | - | - | * | * | * | 5 |
| Hamilton / 1995 | * | - | * | - | - | - | * | * | * | 5 |
| Kane / 1991 | * | - | * | - | - | - | * | * | * | 5 |
| Hefny / 2015 | * | - | * | - | - | - | * | * | * | 5 |
| Faget / 2015 | * | - | * | - | - | - | * | * | * | 5 |

Item 1 = representativeness of cohort study. Item 2 = selection of non-exposed cohort. Item 3 = ascertainment of exposure. Item 4 = outcome not present at start. Item 5 = comparability on most important factor. Item 6 = comparability on additional factor. Item 7 = assessment of outcome. Item 8 = follow-up long enough. Item 9 = adequacy of follow-up. * = one point. - = zero points.

Most included studies were retrospective and scored between 5-7. The lack of comparability adjustments across the studies may influence pooled estimates. Nevertheless, outcome assessment was consistently robust, with most injuries confirmed surgically.


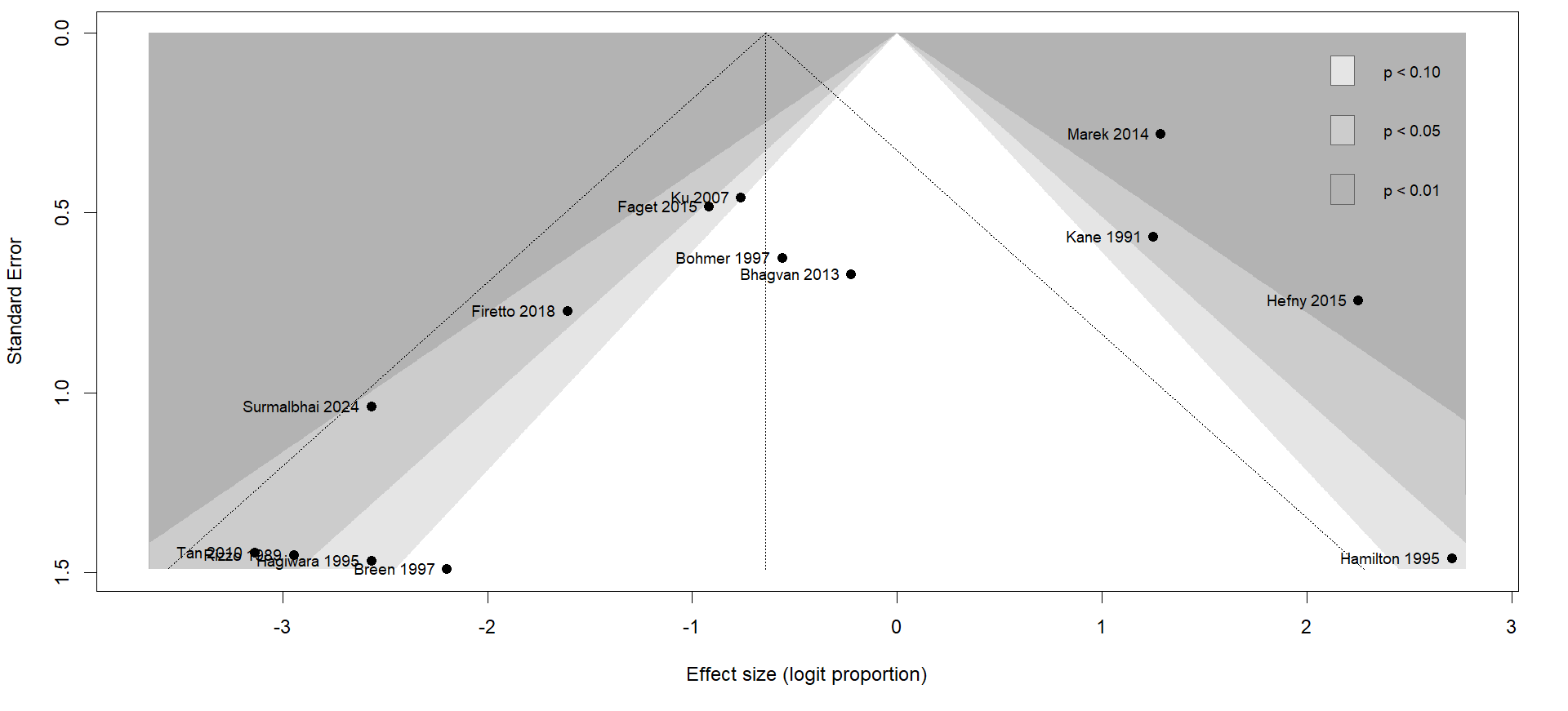


**Figure S1. Funnel plot assessing potential publication bias among studies reporting the proportion of blunt trauma patients with CT-detected free intraperitoneal air who had no significant bowel perforation.**
